# Engineering D-Amino Acid Containing Collagen Like Peptide at the Cleavage Site of *Clostridium histolyticum* Collagenase for Its Inhibition

**DOI:** 10.1371/journal.pone.0124398

**Published:** 2015-05-14

**Authors:** Punitha Velmurugan, Raghava Rao Jonnalagadda, Balachandran Unni Nair

**Affiliations:** Council of Scientific and Industrial Research—Central Leather Research Institute, Chemical Laboratory, Adyar, Chennai, 600 020, India; Russian Academy of Sciences, Institute for Biological Instrumentation, RUSSIAN FEDERATION

## Abstract

Collagenase is an important enzyme which plays an important role in degradation of collagen in wound healing, cancer metastasis and even in embryonic development. However, the mechanism of this degradation has not yet been completely understood. In the field of biomedical and protein engineering, the design and development of new peptide based materials is of main concern. In the present work an attempt has been made to study the effect of ^D^Ala in collagen like peptide (imino-poor region of type I collagen) on the structure and stability of peptide against enzyme hydrolysis. Effect of replacement of ^D^Ala in the collagen like peptide has been studied using circular dichroic spectroscopy (CD). Our findings suggest that, ^D^Ala substitution leads to conformational changes in the secondary structure and favours the formation of polyproline II conformation than its L-counterpart in the imino-poor region of collagen like peptides. Change in the chirality of alanine at the cleavage site of collagenase in the imino-poor region inhibits collagenolytic activity. This may find application in design of peptides and peptidomimics for enzyme-substrate interaction, specifically with reference to collagen and other extra cellular matrix proteins.

## Introduction

Biomolecules are asymmetric in nature, which are predominantly chiral in existence in concern with stereochemistry. These molecules are present in two forms, named as enantiomers, which is important for their function. In these biomolecules, carbon acts as chiral center by holding four non-equivalent substituent and leads to non-superimposable mirror images. E.g., all the amino acids are in L-form in proteins except glycine. Conferring to the D/L naming convention, only one of the two enantiomers is widely used in nature. For example, amino acids and sugars are in L- and D-configuration, respectively. In the past, it has been believed, only L- amino acids are present in the living system. However, D-amino acids are identified in bacterial cell wall [1–3] and in lower animals such as snails [4,5], crustaceans [6], and spiders [7–9] and amphibians skin peptides dermorphin and deltorphin [9–11]. Some of the D-amino acid containing bacterial peptides, dermorphin and deltorphin are related to the mammalian hormones and neurotransmitters. Nevertheless, D-amino acids have recently found in mammals [12] as in free form and as well as a constituent of many peptides and proteins for instance amyloid β peptides [13–16], α-crystallin [17], skin proteins [18] and Australian duck-bill platypus [19,20]. D-amino acids are plays an important role in diseases, such as senile cataract, Alzheimer’s dementia [21], and skin aging [22].

D-amino acid residues in peptides and proteins is known to alter the three dimensional structure and it may provide several uses, such as (i) Enhances their resistance to proteolysis, (ii) Involves in changing the conformational characteristics, (iii) Act as probe to elucidate the relationship between the conformation and its bioactivity, (iv) Design of novel peptides, (v) Act as a signalling molecule in helix termination, and (vi) Possess antibacterial and antitumor activity [23–26]. D-amino acid substitution in α-helices causes only a local change in structure and flexibility at the substituted site, which leads to destabilization subsequently it helps to probe the structure activity relationship between conformational domains and bioactivity. ^D^Pro substitution in β-hairpin peptide acts as a conformational determinant, which helps to form artificial reverse turns as well as ^D^Ala nucleates the formation of β-hairpin.

Racemization and post-translational modification reactions lead to the formation of D-amino acids in proteins [27–28], crystalline [17], and collagen [29–31]. ^D^Asp estimation in collagen is used as a dating tool in biological samples [30–31]. The rate of racemization in collagen varies with respect to amino acid residues and its exposure towards temperature, pH and radiations [32–35]. Collagen is the most abundant protein in human body. Till date 28 type’s isoforms of collagens are identified in the human species. Collagen triple helix is formed by the polypeptides stands, which forms left handed polyproline II conformation. These three identical or non-identical polypeptide strands are inter-twisted each other to form a tightly packed right handed triple helix. Because of size of the collagen molecule it is difficult to study the structure, assembly and its biochemical aspects. Smaller peptides are used to overcome these difficulties faced in the natural collagen. In collagen, polypeptide strands (α-chains) are made up of unique sequential pattern of three amino acid repeat of X_AA_-Y_AA_-Gly, where X_AA_ and Y_AA_ can be any amino acid, most frequently proline and hydroxyproline, respectively [36–38]. Most preferably model peptides with Pro-Hyp-Gly patterns are used to elucidate the folding and structure of collagen. Effect of mutations and interruptions are studied with these peptide repeats [39–54]. Extensive investigations have been done with Pro-Hyp-Gly repeats and studies with the natural sequence are in scarce. As well as studies related to the effect of D-aminoacid in collagen are limited. Gly→D-aminoacid substitution in collagen like peptides leads to destabilization in position and residue dependant manner. Replacement of Gly residue with ^D^Ala and ^D^Ser residues lead to the formation of triple helical conformation than its L-isomers [55]. ^D^Asp substitution instead of its L- isomer prevents the triple helical formation but it favors to form heterotrimer triple-helical molecules under racemic mixture conditions of D- and L-Asp [56]. Our earlier molecular dynamics results reveal that, L→D-aminoacid substitution in Pro-Hyp-Gly repeats leads to the formation of kink at the substituted site [25]. In peptide sequence Furylacryloyl-Leu-Gly-Pro-Ala (FALGPA), ^D^Leu substitution in its L-counterpart inhibits the collagenolysis [24]. In the present work, we have constructed the ^D^Ala substituted triple helical peptide model in the collagenase cleavage site from type I collagen (Uniprot accession number P02452 residues from 935–970) to study the effect of ^D^Ala on collagen conformation and its collagenolytic behaviour of *Clostridium histolyticum* collagenase. As mentioned earlier, D-amino acids in polypeptide sequence are resistive to proteolytic activity and although proteins containing D-amino acids can be hydrolyzed at peptide bonds containing L-amino acids, the hydrolysis rate may be slower than those for corresponding native proteins. The thermal and structural stability of ^D^Ala substituted collagen like peptide and its susceptibility for collagenase have been analyzed.

## Experimental Section

### Materials

Collagen like peptide was customarily synthesized using Fmoc chemistry in advanced protein chemistry laboratory, Tufts University, USA. The synthesized native sequence was from the α_1_ chain of type I collagen in PDB number P02452 residues from 935–970 including Hyp at Y_AA_ position. The constructed collagen like peptide sequence is GSO GAD GP^D^AGAOGTO GPQ GIA GQR GVV GLOGQRGER and its molecular weight is 3385.35 Da. All other chemicals and solvents were used in analytical grade. Amino acids are represented in the single letter codes. Upper case and superscript ‘D’ letters represent L- and D-Amino acid, respectively. The hydroxyproline residue is represented as O. The terminals are blocked with acetyl (Ace) and *N*-methyl (Nme) groups.

### Triple helix formation and characterization

Peptide sample was prepared in aqueous solutions with the concentration of 1 mg/mL. The sample was heated at 60°C for 5 minute then immediately stored at 4°C for a minimum time period of 72 h to allow formation of triple helical conformation.

### Secondary structural analysis- Circular dichroism spectroscopy

Circular dichroism (CD) measurements were conducted on a Jasco J-815 CD spectrophotometer model equipped with a peltier temperature controller-423S/15 (JASCO Inc.). CD spectra of peptide sample was recorded at 4°C in 10 mM tris buffer at pH 7 using 1 mm path length quartz. Wavelength was scanned between 250 to 190 nm at 4°C using 1.0 nm band width, 0.1 nm step size, for an average time of 1s with an average of three sans at a scan speed of 100 nm/min. Data analysis was performed with CONTIN software packages [57–59]. For thermal unfolding measurements, CD in molar ellipticity was monitored at 220 nm as a function of temperature from 4 to 70°C at an average heating of 1°C/min to monitor thermal transitions.

### Dynamic Light scattering (DLS) measurements

DLS measurement was performed in 90-plus nanoparticles size analyser, Brookhaven instruments, with the laser wavelength of 660 nm at a 90° scattering angle equipped with temperature controller. Measurement was conducted at 6°C using 1.5 mL cuvette. Prior to sample measurement, the sample was incubated at 4°C for 72 h. Before the measurement, the sample was filtered through 0.2 μm pore size filters. The hydrodynamic diameter was obtained by analysing intensity autocorrelation function with 90-plus particle size software package attached to the Brookhaven instrument.

### Transmission electron microscopy (TEM)

3 μL of peptide sample placed on a carbon coated copper grid and allowed to dry at room temperature further stained with 2 μL of 1 w/v % phosphotungstic acid solution for 30 s. The excess staining solution was blotted using filter paper and TEM grids were dried at room temperature for two hours prior to imaging. TEM images of peptide were obtained using Hitachi H-7650 at an acceleration voltage of 80 kV at room temperature.

### Enzyme Kinetics

#### Circular dichroism spectroscopy

CD spectra was recorded over the range λ = 250 to 190 nm to analyse the effect of *Clostridium histolyticum* bacterial collagenase on collagen like peptide at pH 7, 37°C for 2 h in 10 mM tris buffer. CD spectra was measured using 1 mm path length quartz with 0.5 mg/mL concentration of collagen like peptide with 1.0 nm band width, 0.1 nm step size, for an average time of 1s with an average of three sans at a scan speed of 100 nm/min. Data analysis was performed with CONTIN software packages [57–59].

#### RP-HPLC measurements

For enzyme hydrolysis, the peptide samples were prepared with and without collagenase at 37°C with 2 h incubation at a peptide concentration of 0.5 mg/mL in Tris buffer in the presence of calcium. After two hours of incubation the samples were subjected to RP-HPLC. RP-HPLC experiments were performed by Agilent 1100 semi preparative HPLC (Agilent Technologies, Santa Clara, CA, USA) equipped with auto sampler and temperature controller using an Agilent Zorbax 300 extend-C18RP, 5 micron particle size (4.6X250 mm). The RP-HPLC experiments were carried out with a linear gradient flow of 0.1% TFA in water and 0.1% TFA in acetonitrile from 10 to 50% in 35 minute for collagen like peptide elution with a flow rate of 1 mL/min. The injection volume for the analysis of peptide is 50 μL of 0.5 mg/mL concentration of peptide. The chromatogram was observed for 35 minute using diode array detector with detection at 214 nm.

## Results and Discussion

We have customarily synthesized the sequence GSO GAD GP^D^A GAO GTO GPQ GIA GQR GVV GLO GQR GER because this imino poor sequence expected to act as a good model to study the collagenolysis. In addition, sequence has GER motif, which is an essential triplet for binding to various integrins expressed on cell surfaces and blood platelets to enhance the cell adhesion activity [60–61]. These charged motifs are thermally stable in the absence of hydroxylproline. Furthermore, Asp in Y_AA_ has the ability to from ion pair interactions [62]. Our earlier molecular dynamic results reveal that ^D^Ala substitution induces lowest destabilization effect on triple helical structure than other D-amino acids with destabilization energy of 7.87 kcal/mol/^D^Ala substitution [25] as well as Ala present in cleavage site of collagenase. Based on the molecular dynamics result and cleavage site behaviour of collagenase, we have substituted the ^D^Ala in the cleavage site of bacterial collagenase in CLP to prevent the proteolysis.

### Conformational analysis using circular dichroism spectroscopy (CD)

**Fig 1:**
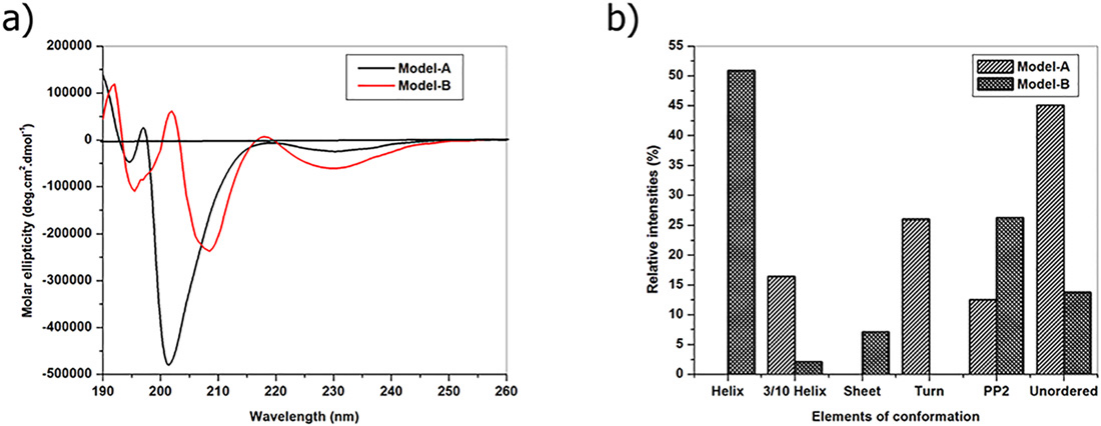
(a) Far UV circular dichroism spectra of native (Model-A) and ^D^Ala substituted (Model-B) collagen like peptide; (b) Calculated secondary structure fractions of peptide conformation from CD data using CONTIN software package.

^D^Ala substituted collagen like peptide show the positive peak at 218nm, negative maximum at 208 nm with shoulder at 230 nm and crossover point at 216.5 nm with the molar ellipticity values of 6625, -236377and -60843 deg.cm^2^.dmol^-1^, respectively. The characteristic wavelength of triple helical conformation at 218 nm shows the molar ellipticity in deg.cm^2^.dmol^-1^ of 6625 suggests that ^D^Ala substitution favours to form triple helix in the imino poor region compared to its L-counterpart. ^D^Ala substituted peptide shows a very low ellipticity in positive maxima with the slight blue shift compared to compact polyproline II conformation (220–222 nm). This indicates that the selected imino poor sequence has a low propensity to form a compact triple helical conformation. Counterpart of the discussed peptide shows doesn’t have ability to form a polyproline II conformation which can be inferred from the absence of positive peak at 220 nm. It is an additional evidence for the favoured stability of the ^D^Ala substitution in selected imino poor regions. Native peptide predominantly present in monomeric strands or not able to form stable compact triple helix in contrast to that ^D^Ala substitution, which favours the formation of the flexible triple helical conformation in the imino poor region. The approximate fractions of each secondary structures conformation peptides and proteins can be estimated by analyzing their CD spectra using multiples of reference spectra for each structural type [63]. In this work the secondary structural elements of collagen like peptide was calculated from CD data at pH 7.0 using CONTIN program and the result has been shown in Fig 1b. It is indicated that ^D^Ala substituted peptide contains the secondary structural elements of 53% helix structures, ~7% sheet, ~26% polyproline II structures and ~14% unordered structures. In the case of its enantiomeric counterpart peptide is leads to the formation of highly unordered conformation. ^D^Ala substituted peptide favours to form helix and polyproline II conformation. Structural analysis suggests that, ^D^Ala substitution forms stable conformation than its L-counterpart in imino poor region.

Circular dichroism spectroscopic measurements were performed to determine the secondary structure of native and ^D^Ala substituted imino poor collagen like peptide. ^D^Ala substituted collagen like peptide show the weak characteristic triple helical state, as well as it shows the similar spectral signature of imino poor bacterial collagen [60–61] and CD spectra have been shown in Fig 1a.

**Fig 2:**
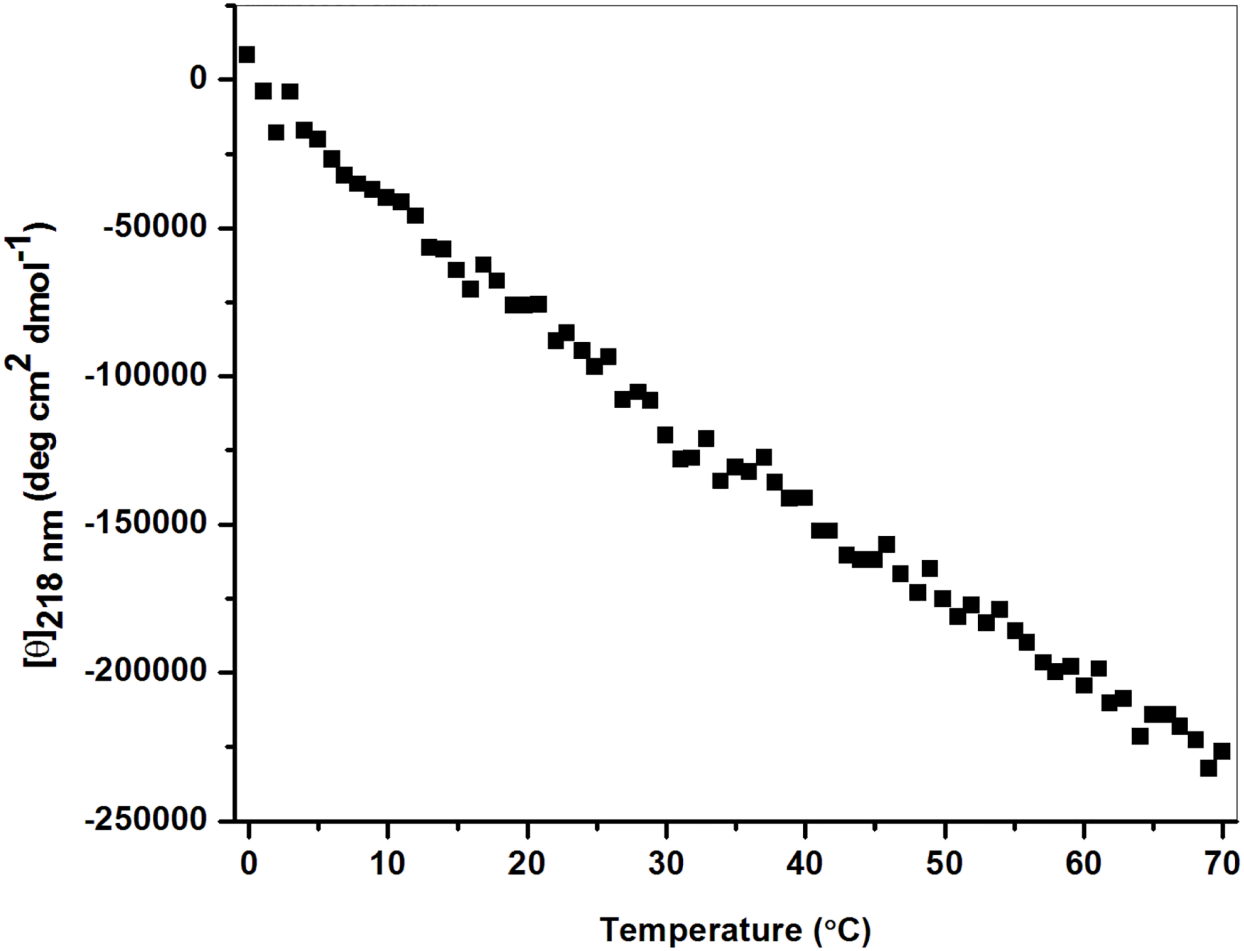
Thermal transition curve for ^D^Ala substituted peptide in 10 mM tris buffer at pH 7.

Peptide denaturation was studied by measuring the changes in molar ellipticity at the wavelength of 218 nm (positive maxima) with increase in temperature, which has been shown in Fig 2. Linear decrease in ellipticity with increase in temperature reveals that peptide denaturation falls in monotonic manner rather than cooperative transition. Cooperative transition is the characteristic unfolding nature of collagen but the selected sequence results in monotonic transition. It suggests that the ^D^Ala substituted collagen like peptide fails to form stable triple helical conformation which agrees with several authors, because at least of six triplets of GPO is necessary to form stable triple helix [52,64–67]. Melting profile of the ^D^Ala substituted peptide is similar to its L-counterpart (S1 Fig) because the selected peptide sequence lacks in GPO repeats.

### Investigation of self assembling behaviour of peptide usingdynamic light scattering spectroscopy (DLS) and transmission electron microscopy (TEM)

**Fig 3:**
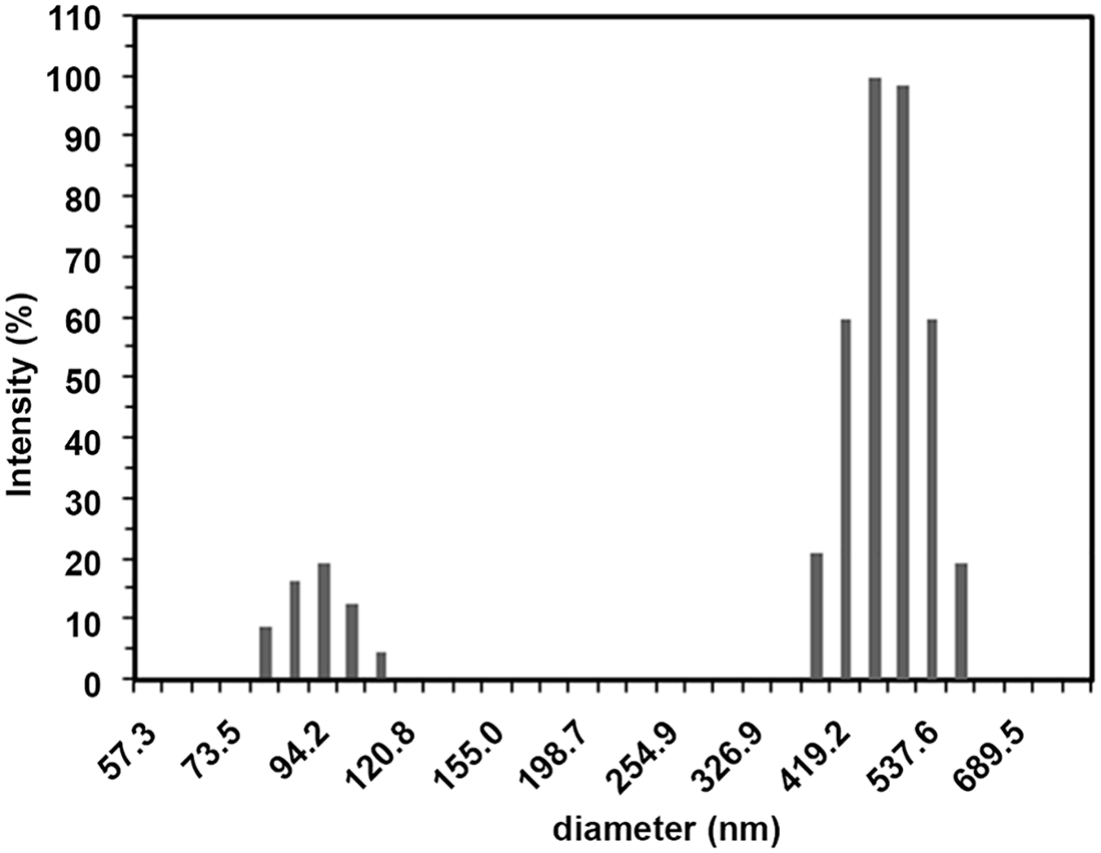
Size distribution analysis of peptide solution in 10 mM Tris buffer at pH 7 via dynamic light scattering.

**Fig 4:**
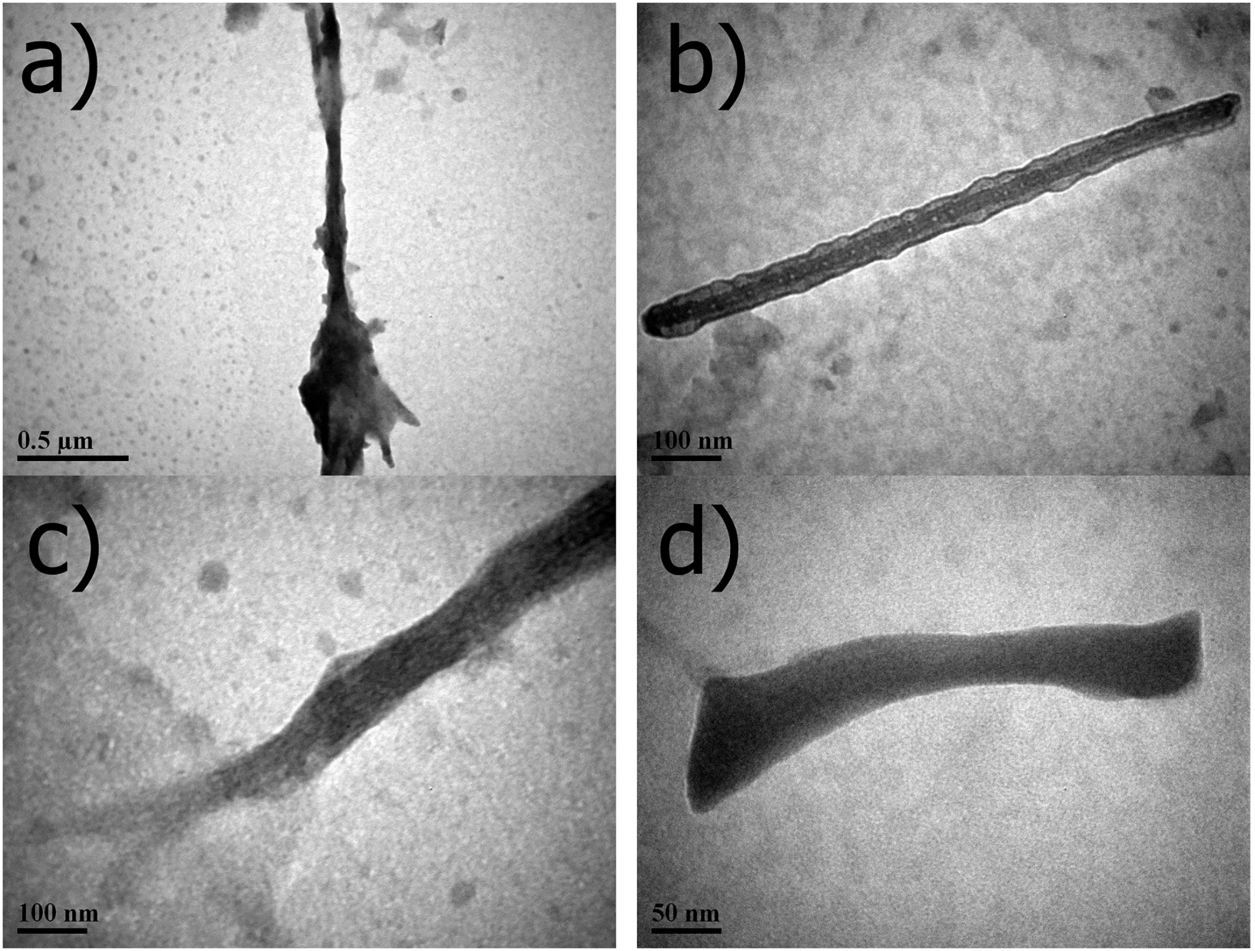
TEM images of peptide assemblies stained with phosphotungstic acid on nano- and micro-scales. Scale bar represents for (a) 0.5 μm; (c) and (d) 100 nm; (e) 50 nm.

Collagen is a hierarchical protein with different in supramolecular assembly which provides the mechanical and functional roles in tissues. Size distribution of peptide assemblies were recorded from DLS in terms of intensity versus hydrodynamic diameter and distribution profile has been shown in Fig 3. Hydrodynamic profile shows the effective diameter of peptide ranges from 80 to 110 nm and 385–584 nm with the mean and effective diameter of 420 and 355nm, respectively. The observed hydrodynamic diameters are mainly due to self-assembly and aggregation behaviour of peptide because particles with sizes greater than 10 nm indicates the formation of larger order assemblies, while the small particles are attributed to the soluble triple helix monomer or single chain. To investigate the morphology of the assembled structures peptide was further characterized via TEM. Fig 4 represents TEM images of the imino poor region of the collagen, which revealed the presence of supra-molecular assemblies existing as agglomerated sponge like structures, rods and unordered assemblies. Morphologies of the self-assembled peptides is reliable with the previous studies of collagen mimetic peptides and also images showed regular rod shaped structures except that micro level sponge like structures which may be due to agglomeration of the self-assembled structures. In supra-molecular assembly of peptides rods are observed with average diameter and lengths of 50–150 nm and 300–500 nm, respectively. The observations of the length scales of peptide assemblies are consistent with the previous studies. Kotch et al., reported the fibrils whose dimensions are 30–400 nm × 0.5–1.0 nm [68] and Cejas et al., reported the fibril with an average diameter of 0.26 μm [69]. Przybyla et al., shows extensive branching and unbranched region of peptide, which show the bundles of thinner fibrils [70]. Krishna et al., observed the nano rods dimensions are smaller than 60 × 5.5 nm and micro fibrils with the dimensions whose greater than 6 μm × 130 nm for the hydroxyproline lacking collagen mimetic peptides [71]. The peptide exhibits larger self-assembly, because self-assembling behaviour and formation of triple helix starts at the C-terminal [72–74]. These sequences have ability to form loose triple helix, which can form self-associated hierarchical structures or disordered into unfolded unordered form.TEM results of nano rods and micro fibrils results are consistent with the DLS observations.

### Investigation of collagenolysis behaviour using CD and reverse phase high performance liquid chromatography (RP-HPLC)

**Fig 5:**
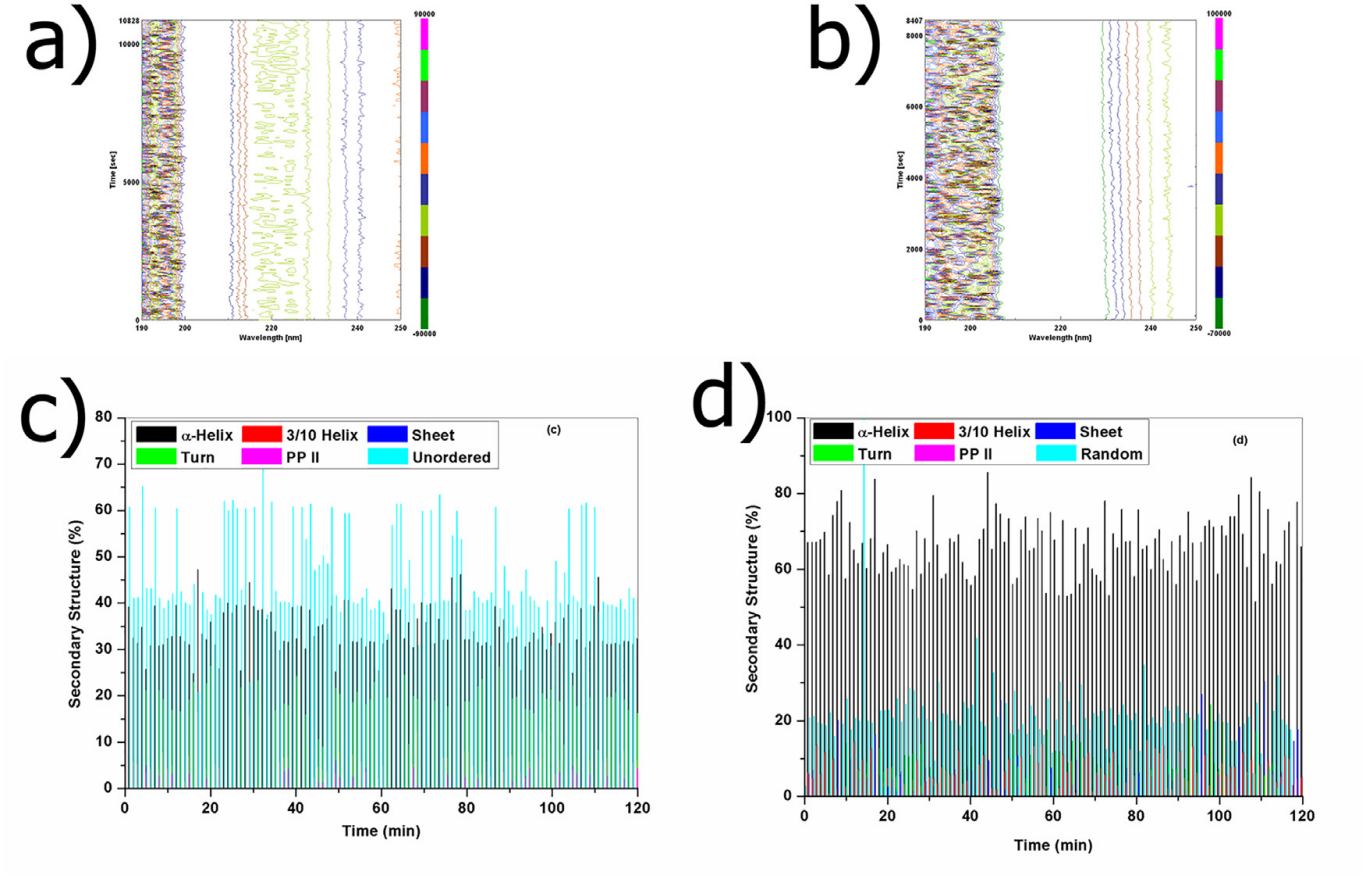
(a) Change of CD spectral signature of ^D^Ala substituted peptide with increase in time from 0 to 120 min at 37°C in 2D-view; (b) Change of CD spectral signature of ^D^Ala substituted peptide upon interaction with bacterial collagenase with increase in time from 0 to 120 min at 37°C in 2D-view (c) Relative amount of different structural elements in ^D^Ala substituted collagen like peptide with increase in time from 0 to 120 min at 37°C using CD data by CONTIN software package; (f) Relative amount of different structural elements in ^D^Ala substituted collagen like peptide upon interaction with bacterial collagenase with increase in time from 0 to 120 min at 37°C using CD data by CONTIN software package.

CD spectroscopic measurements were performed to evaluate the extent of conformational changes of collagen like peptide upon the interaction with bacterial collagenase. Change of CD spectral signature with increase in time was recorded at 37°C which has been shown in Fig 5a and 5b. Drastic structural changes have been observed in the spectral region of 225–215 nm for the peptide with increase in time at 37°C. Interaction of bacterial collagenase with the peptide leads to drastic structural changes in the spectral region of 205–190 nm without collagenolysis. Binding of bacterial collagenase with the ^D^Ala substituted peptide induces secondary structural changes in the imino poor region, which has been observed from the CD spectral signature. Substitution of ^D^Ala in the imino poor region in the cleavage site of collagenase restricts the collagenolysis. L→D configurational change in cleavage site of collagenase helps to understand the effect of binding of collagenase on collagen.

**Fig 6:**
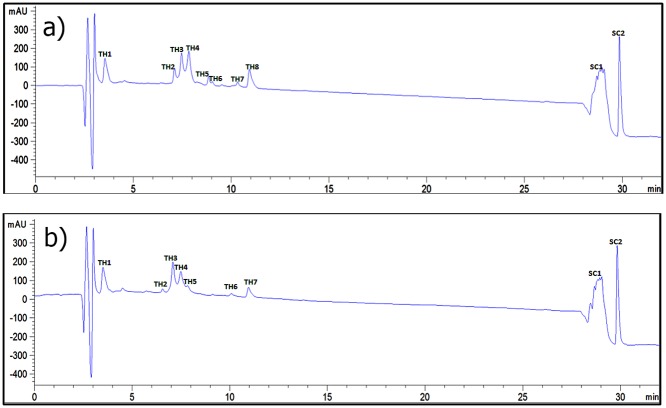
HPLC chromatogram for (a) ^D^Ala substituted imino poor region of type I collagen at 37°C; (b) ^D^Ala substituted imino poor region of type I collagen after 2 h incubation with bacterial collagenase at 37°C.

Separation of biological molecules in RP-HPLC varies with respect to its hydrophobicity. Hydrophobicity of the collagen like peptide is mainly depends on the X_AA_ and Y_AA_ amino acids in Gly-X_AA_-Y_AA_ repeats which are responsible for the hydrophobic and hydrophilic character of triple helix, because X_AA_ and Y_AA_ positioned amino acids are point outward. Enzyme hydrolysis has been monitored using RP-HPLC with the detection at 214 nm, 37°C and Chromatogram has been shown in Fig 6 and S2 Fig Formation of triple helical conformation can be confirmed by the formation of new peaks using RP-HPLC with the detection at 214 nm. Chromatogram shows multiple peaks in which shorter retention time peaks have been assigned as triple helix (TH) and longer retention time peaks have been assigned as Single chain (SC). Chromatogram of peptide shows multiple peaks, which represents the triple helical conformation. This may be due partial folding of peptides or misalignment within the triple helical structure during the nucleation process or single chain to triple helix process [75]. Shorter retention time peaks has been denoted as TH1 to TH 9 and longer retention time peaks has been assigned as SP1 to 2. After incubationwith bacterial collagenase for 2 hours shows similar peaks, which indicates no collagenolysis has been observed. Asmall shift in retention time has been observed in the chromatogram, which may be due to conformational change in peptide upon collagenase binding leads to change in hydrophobicity.

## Conclusion

This work elucidates the effect of ^D^Ala in imino poor region of type I collagen molecule on collagenolytic behaviour through the detailed study of secondary structural, thermal and enzymatic stability. Structural analysis reveals that ^D^Ala substitution in the imino poor region favours to form triple helix than its L-counterpart. ^D^Ala substitution in the imino poor region forms stable conformation than the native. The TEM and DLS analysis suggest that, ^D^Ala substituted imino poor region involves in the self-assembling and aggregation behaviour. Substitution of ^D^Ala in the cleavage site of collagenase restricts the collagenolysis of *Clostridium histolyticum* bacterial collagenase. We anticipate that these ^D^Ala substituted collagen like peptides will help in new collagen based biomaterials hold great potential for biomedical and tissue engineering applications.

## Supporting Information

S1 Fig Thermal transition curve for native peptide in 10 mM tris buffer at pH 7. Molar ellipticities were recorded at λ = 220 nm while the temperature was increased from 0 to 70°C.
(TIF)

S2 Fig HPLC chromatogram for native peptide at 37°C (a) without collagenase, (b) with collagenase (TIF)
