# Supplementary figures and images for: Engineering D-Amino Acid Containing Collagen Like Peptide at the Cleavage Site of Clostridium histolyticum Collagenase for Its Inhibition
Source: PLoS One. 2015 May 14;10(5):e0124398. doi: 10.1371/journal.pone.0124398 (PMC4431724; doi:10.1371/journal.pone.0124398)

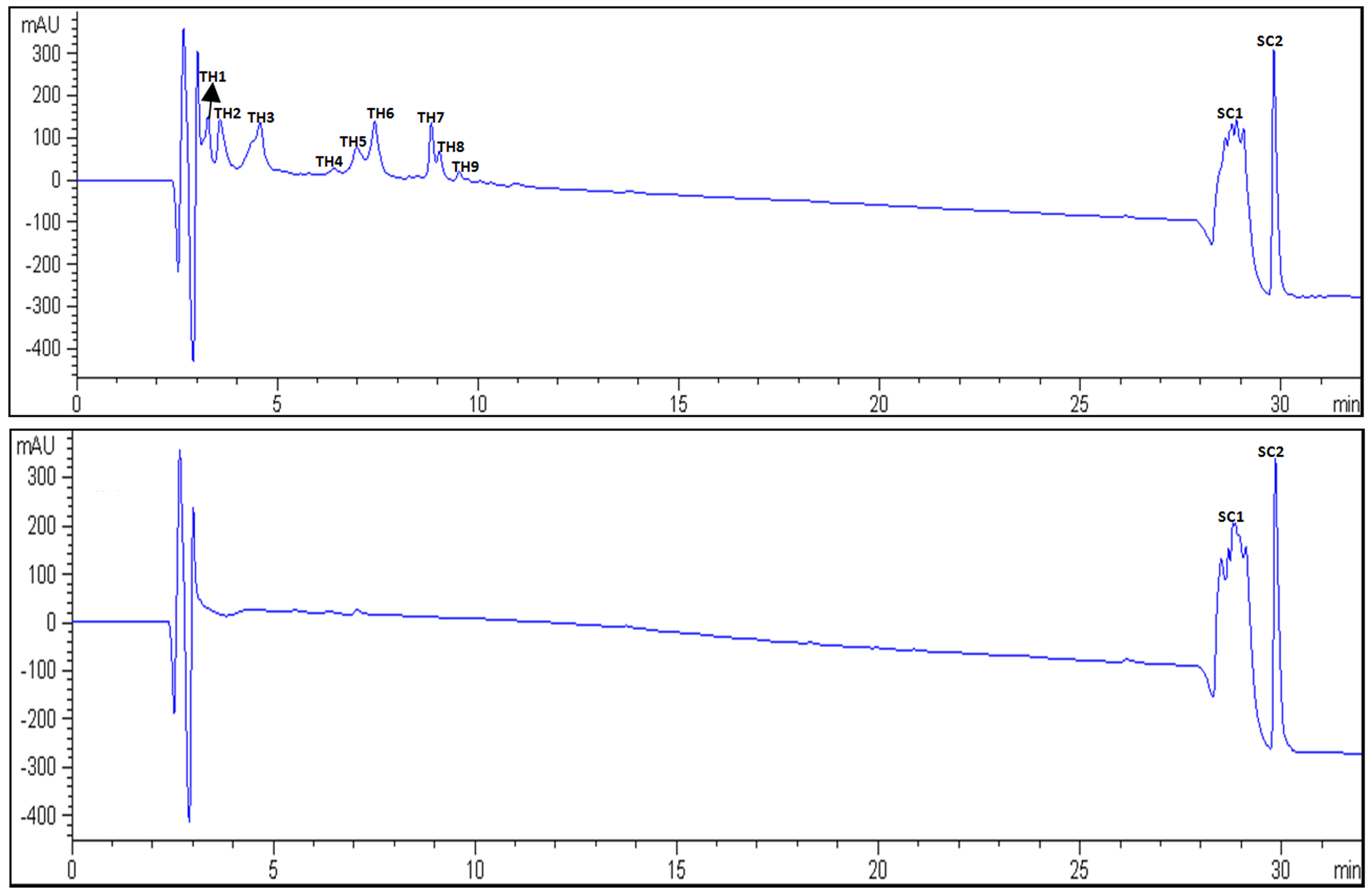

Supplement: S2 Fig — (TIF) [file pone.0124398.s002.tif]
